# Horizon: A Trajectory Optimization Framework for Robotic Systems

**DOI:** 10.3389/frobt.2022.899025

**Published:** 2022-07-13

**Authors:** Francesco Ruscelli, Arturo Laurenzi, Nikos G. Tsagarakis, Enrico Mingo Hoffman

**Affiliations:** ^1^ Humanoids and Human Centered Mechatronics (HHCM), Istituto Italiano di Tecnologia (IIT), Genova, Italy; ^2^ PAL Robotics, Barcelona, Spain

**Keywords:** trajectory optimization, legged robotics, non-linear programming (NLP), locomotion, motion planning

## Abstract

This paper presents *Horizon*, an open-source framework for trajectory optimization tailored to robotic systems that implements a set of tools to simplify the process of dynamic motion generation. Its user-friendly Python-based API allows designing the most complex robot motions using a simple and intuitive syntax. At the same time, the modular structure of Horizon allows for easy customization on many levels, providing several recipes to handle fixed and floating-base systems, contact switching, variable time nodes, multiple transcriptions, integrators and solvers to guarantee flexibility towards diverse tasks. The proposed framework relies on direct simultaneous methods to transcribe the optimal problem into a nonlinear programming problem that can be solved by state-of-the-art solvers. In particular, it provides several off-the-shelf solvers, as well as two custom-implemented solvers, i.e. GN-SQP and Iterative Linear-Quadratic Regulator. Solutions of optimized problems can be stored for warm-starting, and re-sampled at a different frequency while enforcing dynamic feasibility. The proposed framework is validated through a number of use-case scenarios involving several robotic platforms. Finally, an in-depth analysis of a specific case study is carried out, where a highly dynamic motion (i.e., a twisting jump using the quadruped robot Spot^®^ from BostonDynamics^1^) is generated, in order to highlight the main features of the framework and demonstrate its capabilities.

## 1 Introduction

The versatility of floating-base robots, such as legged, wheeled and, more in general, articulated platforms, pushes forward the boundaries of robot capabilities. Motion planning exploits this potential to impose a desired behaviour in terms of kinematic and dynamic references for the robot. In the scenario of agile, highly dynamic and contact-rich motions, this goal corresponds to finding feasible trajectories for under-actuated complex systems subject to non-linear, hybrid dynamics. Indeed, as designing these trajectories by hand proves burdensome, different strategies to compute them can be found in literature. Among the others, one powerful approach is *optimal control*: seeking for a trajectory governed by the system dynamics that satisfies some desired constraints, boundary conditions and minimizes a given performance index. This formulation allows to specify high-level task behaviours as a combination of simple objective and constraint functions, letting the dynamics of the system, and the desired constraint, shape the resulting motion to guarantee feasibility and physics consistency. This is especially critical in any complex scenario that involves locomotion and fast interactions with the environment, as it is crucial to generate feasible trajectories. Furthermore, through this formulation, the complexity of the robot model can be fully exploited: full robot kinematic and dynamic models, as opposed to simplified templates, allows to specify a vast range of constraint and cost functions, that translates into more complex motions, pushing the limit of what the hardware can accomplish in terms of speed and strength.

Finding a solution to this family of optimal control problems is often computationally heavy and cannot meet the frequency requirements to run in a control loop. On the contrary, linearizing around a nominal trajectory allows for fast computation, but the validity of the result decreases moving away from the region of state space where the nonlinear dynamics were linearized. This approach is useful when a nominal trajectory is found, as obtaining that trajectory may imply traversing a highly non-linear state space. The sub-problem of *trajectory optimization* only considers the open-loop solution to optimal control. Indeed, if the problem can be solved at a desired rate, it can be used to iteratively generate a closed-loop trajectory for the robot. An effective tool to rapidly generate robot trajectories should provide the user with a pipeline encompassing the whole problem of loading a robot model, imposing the desired behaviour, and obtain a dynamically-feasible motion that satisfies the user’s instructions. In doing so, it should supply an interface and methods relevant to robotics applications (such as forward and inverse dynamics). Furthermore, is should offer the possibility to re-compute and send reference trajectories in a receding horizon fashion.

**FIGURE 1:**
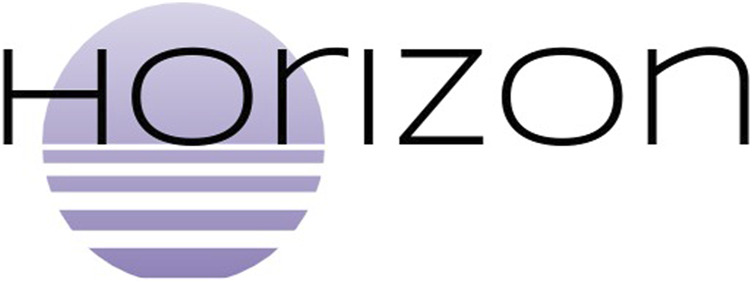
The Horizon framework is available at: https://github.com/ADVRHumanoids/horizon.

Given these requirements, this work introduces Horizon (Figure 1), a trajectory optimization framework designed specifically for robotic systems, that allows to setup and solve problems in both an offline and receding horizon fashion, seeking to simplify the pipeline for optimal motion planning without shadowing the underlying mechanics. Horizon provides a high-level syntax that covers from model description to problem formulation and solution post-processing: it is organized in transparent modules, so that ease-of-use does not undermine ease-of-access: it exposes each layer so that it can be easily customized to the user needs. Nevertheless, thanks to the intuitive syntax, the non-expert user can treat Horizon as a black-box to generate desired trajectories for any given fixed or floating-base robot. The contribution of this work lies in the development of a complete pipeline consisting of four main building-blocks:

• *Robot model acquisition*, i.e. the parsing of a URDF model, in a way the exposes standard robot algorithms to the user.
• *Problem definition*, i.e. a concise syntax to specify costs and constraints, and distribute them over the planning horizon.
• *Solver selection*: different solvers are available to meet different requirements, as detailed in Section 4.4.
• *Receding horizon* formulation, thanks to tools for cheaply relocating costs and constraints to different nodes, to accommodate for a receding horizon scenario.
• *Optimal trajectory post-processing*, i.e. methods to re-sample the trajectory at a given frequency, and perform *mesh refinement* to meet physics constraints between time knots.

Horizon leverages on the *CasADi* framework (Andersson et al., 2019), taking advantage of state-of-the-art automatic differentiation techniques for derivatives computation, as well as the *Pinocchio* library for robot kinematics and dynamics (Carpentier et al., 2019), to provide standard algorithms in a symbolic form to be compatible with the CasADi computations. The resulting framework collects the useful tools to prototype highly dynamic maneuvers in a simple and intuitive way. Horizon was successfully tested to generate a vast range of behaviours using several robots, as it support fixed and floating-base models: from simple template models of legged robots to full models of quadrupeds and humanoids. It is worth mentioning that, thanks to the fully generic symbolic layer provided by CasADi, Horizon can be used even outside the scope of robotics. Besides the above-mentioned functionalities, Horizon was conceived with the following key properties in mind:

• *Robotics focus.* The toolbox targets robotic applications by providing built-in methods and robotics-oriented utilities. The user should be able to choose from a broad selection of standard algorithms that can use to define system dynamics given state and input variables and generate constraint and cost functions, such as inverse dynamics, under-actuation and friction cone bounds.
• *User-friendly interfaces.* The syntax to build the optimization problem should be as intuitive as possible. As the construction of an optimal control problem tends to be tedious and verbose, Horizon offers an environment that simplifies the formulation process. Also non-expert users, lacking an in-depth knowledge of optimization algorithm, should be able to set up an optimization task to prototype complex motions with minimal effort. Experienced user, on the other side, should be able to access easily each module of the toolbox for customization.
• *Open-source.* The software is based on open-source packages, and should be freely available itself. Any user should be allowed to experiment with the code and propose useful modifications.
• *Flexibility and compatibility.* Each software modules should be accessible and extendable to grant two valuable properties: first, the modularity of Horizon allows to integrate it in other pipelines avoiding code duplication. Second, each module can be extended with custom methods for personalized optimization routines. Horizon promotes compatibility with other frameworks requiring minimal inputs, as the toolbox is self-contained. The only requirements are standard inputs such as XML files (e.g., URDF) to simplify the integration with other projects.
• *Comprehensive pipeline.* The framework should encompass the trajectory generation problem as a whole, without relying on supplementary packages. The user should be able to generate a complete robot motion starting from a simple description of the model used. Horizon is generic enough to include all the necessary tools to prototype a dynamic manoeuvre, optimize a robot structure or produce a walking gait without losing the advantages of a robotics oriented software.

Before delving into the details of proposed framework in Section 4, this paper offers a background on optimal control and non linear programming (NLP) in Section 3. To better present the capabilities of Horizon, a case study and several applications are explored in Section 5. Finally, Section 6 discusses pro and cons of Horizon and proposes future perspectives of development.

## 2 Related Works

Recently, more and more behaviours of legged robots demonstrated unprecedented agility and dexterity: among many approaches to achieve these motions, Horizon joins the increasingly large category of trajectory optimization frameworks. This branch has become widespread among the legged robotics community to specify dynamically feasible motions for articulated robots, on one side for the ever-rising complexity of the tasks and the advancements, on the other for the advent of libraries that can solve non-linear problem very efficiently. Trajectory optimization proved to be a powerful tool to design dynamic motions for linear or nonlinear dynamical systems, especially considering the physical constraints induced by the nature of the manoeuvre, the environment and the hardware itself. Many framework gathered interest within this research topic. While sharing similar underlying strategies to solve non-linear problems numerically, an increasing selection of different framework are available to tackle the problem of designing agile motions, exploiting fully the capabilities of the robot and discovering feasible motions. Several packages have been developed to provide an integrated pipeline involving modeling, planning, and even simulation tools for complex robotic systems. Similarly to *Horizon*, the project *Tropic* (Fevre et al., 2020) leverages the open-source CasADi (Andersson et al., 2019) software, which simplify the construction of optimal control problems. However, it is specific for the generation of bipedal gaits, i.e. joint coordination that minimizes a given cost function along a periodic orbit, resulting in a cyclical walking. Analogous to Tropic, FROST (Hereid and Ames, 2017) is a trajectory optimization toolbox focusing on dynamic locomotion developed in MATLAB. Horizon differs from these specialized frameworks as it is meant to synthesize generic manoeuvres that are non cyclic in nature, and the periodicity can be always injected by imposing appropriate constraints to the problem. Other less-specialized open-source robotic toolboxes, similarly to Horizon, are capable of a more diversified range of motions. Crocoddyl (Mastalli et al., 2020) is a framework for optimal control. While being a powerful tool, its Differential Dynamic Programming (DDP) formulation currently doesn’t handle constraints. Even though some workarounds exist (such as barrier functions), this may be limiting, especially considering that many common constraints in legged robot applications are expressed as inequalities, such as friction cones, kinematic constraints (joint position, velocity, acceleration) and dynamic constraints. OCS2 is a toolbox tailored to switched systems (Farshidian et al., 2017): similarly to Horizon, it facilitates optimal control for robotic tasks, both offline and in a receding-horizon fashion. Furthermore, it also provides an extensive library for model predictive control (MPC). Its core formulation is based on a time-triggered sequence of optimal controls that assumes a fixed sequence of switching modes, such as a predefined gait pattern. Another viable candidate is The Control Toolbox (Giftthaler et al., 2018a), a highly efficient open source C++ library for robotic applications that encompasses modeling, control, trajectory optimization and MPC: in fact, while it can be used for rapid prototyping, its strong focus on efficiency also allows for online operation. However, it is currently only scarcely maintained according to the authors. Among these robot-oriented libraries it is worth mentioning the open-source project TOWR (Winkler et al., 2018), which focus on trajectory generation for legged robots. A powerful software suite that contains a pipeline for trajectory optimization is DRAKE (Tedrake, 2019), on top of a collection of tools for the analysis and the control of robots. DRAKE, similarly to Horizon, exploits automatic differentiation to compute gradients, unlike FROST that uses symbolic computation to construct the NLP solver. Shifting to more general-purpose projects, a relevant candidate is ACADO (Houska et al., 2011), a software environment providing tools for automatic control and dynamic optimization. Other notable toolboxes can be found in literature, but many are commercial, such as GPops (Patterson and Rao, 2014), ForcesPro (Zanelli et al., 2017), MUSCOD-II (Kuhl et al., 2001), DIRCOL (von Stryk, 1999) or Psopt (Becerra, 2010). However, being general-purpose, these packages often lack suitable interfaces for robotics applications (i.e. no robot model construction) and have a low degree of flexibility when constructing the optimal problem (i.e. only one type of transcription is provided).

Table 1 summarizes the main features of a selection of toolboxes compared to Horizon. Each of the above-mentioned packages have proven effective for a specific class of optimal problems. In choosing the most suitable toolbox, there exists two relevant properties to take into account: task and approach specialization. The first indicates how general-purpose the package is, i.e. a formulation generic enough to support a broad spectrum of applications versus a structure specialized for one specific task only. The second points to the degree of flexibility with respect to the underlying algorithms, i.e. a structure tailored to a specific method aiming at performance versus a modular design that favours adaptability. Horizon stands in the middle ground: while it is not general-purpose, as it is strongly tailored to robotics, it is not overly specific for one single task, such as periodic gait generation, and it can be used to generate any kind of motion. On the other side, its modularity allows to choose from a vast range of algorithms and mix them together to better suit a desired goal. Several robotics applications demonstrated the capabilities of CasADi as an effective tool to plan dynamic and contact-rich motions, such as in Mercy et al. (2016), Belli et al. (2021), Hoffman et al. (2021), Nguyen and Nguyen (2021). The aim of this project is creating a robotics-oriented open-source software environment leveraging CasADi strengths that unify the tools to develop these applications in an intuitive way.

**TABLE 1:** Comparison between state-of-art libraries for trajectory optimization and optimal control.

Name	Transcription Method	NLP-Solver	Language	Scope
Horizon	dms, dc	CasADi solvers, ILQR, GN-SQP	Python	generic
DRAKE	dms, dc	IPOPT, custom solvers	C++	generic
Crocoddyl	dms	DDP	C++	generic
OCS2	dms	DDP, SQP	C++	generic
Control Toolbox	dms	IPOPT, SNOPT, ILQR	C++	generic
FROST	dc	IPOPT, SNOPT, Fmincon2	MATLAB	gait generation
TROPIC	dc	CasADi solvers	C++	gait generation
TOWR	dc	IPOPT, SNOPT	C++	locomotion

## 3 Background

Before delving into the description of the framework, we offer an overview of the relevant topics used in the building blocks of Horizon.

### 3.1 Robot and System Dynamics

Horizon is specifically tailored to robotics: while its core functionalities can be exploited for different applications, the entire framework is designed to facilitate the design of optimal problems for robotic platforms. We therefore consider a generic floating-base robotic system and its generalized coordinates $q\in SE\left(3\right)\times R^{n_{j}}$ :

$$q=\left[\begin{array}{c} p\\ \rho \\ \theta \end{array}\right]$$ (1)

where *n*_*j*_ denotes the number of joints composing the robot, $p\in R^{3}$ the linear position of the floating-base, ***ρ*** ∈ *SO*(3) the orientation of the floating-base represented as a unit quaternion, and $\theta \in R^{n_{j}}$ finally represents the vector of joint positions. Given this parametrization, the number of parameters that are needed to describe a configuration is *n*_*q*_ = 7 + *n*_*j*_. We denote the generalized velocities $\nu \in R^{n_{v}}$ as:

$$\nu =\left[\begin{array}{c} \dot{p}\\ \omega \\ \dot{\theta } \end{array}\right]$$ (2)

with *n*_*v*_ = *n*_*j*_ + 6 number of total degrees of freedom in the robot model, $\dot{p}\in R^{3}$ and $\omega \in R^{3}$ the linear and angular velocity of the floating base, and $\dot{\theta }\in R^{n_{j}}$ the vector of joint velocities. The general equation of motion of robot control systems is the following:

$$S\tau =M\left(q\right)\dot{\nu }+h\left(q,\nu \right)-J_{c}^{T}\left(q\right)f_{c}-J_{m}^{T}\left(q\right)\lambda$$ (3)

with $\tau \in R^{n_{a}}$ the vector of actuated joint torques, $M\in R^{n_{v}\times n_{v}}$ the inertia matrix, $h\in R^{n_{v}}$ the non-linear bias terms accounting for gravity, Coriolis and centrifugal torques, $J_{c}\in R^{n_{c}\times n_{v}}$ the contacts Jacobian and $f_{c}\in R^{n_{c}}$ the vector of contact forces, and finally $J_{m}\in R^{n_{m}\times n_{v}}$ is a constraint Jacobian with $\lambda \in R^{n_{m}}$ representing constraint forces to take into account further effects that may be present in the model, for example closed linkages. The selection matrix $S\in R^{n_{v}\times n_{a}}$ is used to map actuated torques to the full vector of efforts in (Eq. 3). Note that usually, the inequality *n*_*a*_ < *n*_*v*_ holds, and *n*_*a*_ = *n*_*j*_ is the common case for fully-actuated floating-base robots. Furthermore, if point-contacts are assumed, then *n*_*c*_ = 3 ⋅ *c* where *c* is the number of contact points. Assuming the following state space representation

$$x=\left[\begin{array}{c} q\\ \nu \end{array}\right],$$ (4)

the following state dynamics equation holds, i.e.

$$\dot{x}=\left[\begin{array}{c} \dot{q}\\ \dot{\nu } \end{array}\right].$$ (5)

In the above equation, $\dot{q}$ is given by the so-called *quaternion propagation* as in Graf (2008)^3^:

$$\dot{\rho }=\left[\frac{1}{2}\omega ,\;0\right]◦\rho .$$ (6)

Note that in (Eq. 60 the symbol ◦ is used to denote the quaternion product.

### 3.2 Optimal Control

Trajectory optimization using NLP formulation is a powerful tool for planning locally optimal trajectories of nonlinear dynamical systems. Given 1) a set of differential equations, which determines the evolution in time of the non-linear system of interest,^2^) initial conditions $x_{0}\in R^{n}$ , and^3^) a vector of static parameters $p\in R^{n_{p}}$ , trajectory optimization aims to design a finite-time input trajectory, $u\left(t\right)\in R^{m}\;\forall t\in \left[t_{0},\;t_{f}\right]$ , which minimizes some cost function over the resulting input and state trajectories $x\left(t\right)\in R^{n}$ , and satisfies a set of constraints. The resulting problem can be formulated as an objective function:

$$J=\int_{t_{0}}^{t_{f}}\ell \left(x,u;p,t\right)\;\text{d}t+\ell_{f}\left(x_{f};p,t_{f}\right),$$ (7)

where the terms *ℓ* and *ℓ*_*f*_ are the intermediate and final costs respectively, subject to the dynamics of the system:

$$\dot{x}=f\left(x,u;p,t\right)$$ (8)

and a set of arbitrary bounds and constraints:

$$\phi^{\min}\leq \phi \left(x,u;p,t\right)\leq \phi^{\max}.$$ (9)

As it is well-known, an optimal problem usually has multiple local solutions as opposed to a single global minimum. In this scenario, any solution [**x**∗(*t*), **u**∗(*t*)] is only guaranteed to be locally minimizing, whereas any valid solution must satisfy all constraints up to a user-defined tolerance.

Besides the very special case where the dynamic system is linear and the cost is described exclusively by quadratic functions, that can be solved exactly using the infinite-horizon linear quadratic problem, a continuous problem (infinite dimensional) must be reduced into a tractable form that can be solved numerically, as described in Section 3.4.

### 3.3 NLP Formulation With CasADi

The backbone of the optimization module is CasADi, an open-source software for nonlinear optimization and algorithmic differentiation Andersson et al. (2019). It provides a set of building blocks to set up and solve efficiently optimal control problems. The key feature of CasADi is its symbolic framework and a state-of-the-art implementation of algorithmic differentiation (AD). As introduced in 3.2, computation of derivatives through numerical differentiation necessarily introduces round-off errors or truncation errors and can become expensive, especially for large problems with a large number of independent variables. In CasADi, objective functions and constraints are defined using symbolic expressions. However, instead of being treated as computer algebra systems (using tree structures), these expressions are stored as directed acyclic graph (DAG) representing mathematical operations: single value or matrix operations, depending on the expression type used in CasADi. Symbolic manipulation guarantees exactness of the solution, but is usually computationally expensive. Automatic differentiation (AD), instead, efficiently computes a derivative calculation by splitting it into a sequence of atomic operations using the chain rule, but applied to floating point numerical values rather than to symbolic expressions, without constructing long symbolic expressions for derivatives. Finally, CasADi exploits efficiently the natural structure of trajectory optimization problems leveraging the graph coloring approach of Gebremedhin et al. (2005) to generate a sparsity pattern that heavily reduces computation times. CasADi is chosen for its ease-of-use, both in Python and C++, its flexible symbolic framework and its compatibility with a number of state-of-the-art solvers, such as SNOPT (Gill et al., 2005), KNITRO (Byrd et al., 2006), IPOPT (Wächter and Biegler, 2006), OSQP (Stellato et al., 2020) and QPOASES (Ferreau et al., 2014). Furthermore, as demonstrated in Fevre et al. (2020), CasADi proves to be efficient, both in time and memory, when solving high-dimensional optimal control problems.

### 3.4 Transcription Methods

In optimal control literature, there are many strategies that can be employed to find a solution. Numerical methods are divided into two broad categories: direct and indirect methods. Planning optimal trajectories of robotic systems mainly relies on the former for transcribing the optimal control problem into a NLP. Direct methods involve the discretization of state and control in accordance to a desired formulation, which defines how to convert the optimization problem into a concrete program that can be managed by solvers.

In literature, the most successful variants for transcription are the simultaneous methods: *multiple shooting* (Bock and Plitt, 1984) and *direct collocation* (Hargraves and Paris, 1987). It is worth noticing that single shooting methods exist, but for complex problem dealing with non-linear systems they are very inefficient. The shortcoming of these method is its inherent high sensitivity to errors. In fact, small changes introduced early in the trajectory can propagate into drastic error near the end. Simultaneous methods (multiple-phase form) lead to a discretization of the time domain as a grid of *N* nodes:

$$t_{i}=t_{0}<t_{1}<\cdots <t_{N}=t_{f}$$ (10)

This implies the parametrization of the trajectory into a vector **w** of optimization variables along the *N* nodes.

Parametrizing the optimal problem requires strategies for a correct transcription: the trajectory is divided into a finite number of segments, connected by junction nodes, and some gap constraints are required to enforce dynamic feasibility by linking each segment. Each node corresponds to a set of variables that can be used to define costs and constraint functions: in other words, the time domain is divided into unconstrained portions and constrained points. Indeed, this strategy leads to one drawback: feasibility conditions may be violated between the grid points, as constraints are enforced only on the nodes and not along the intervals. Then, the problem of finding a continuous optimal trajectory is transcribed into the tractable problem of finding a set of decision variables:

$$\underset{w}{\min}\overset{N-1}{\underset{k=0}{\sum }}\ell_{k}\left(w_{k}\right)+\ell_{f}\left(w_{k}\right)$$ (11)

subject tosystem dynamics (12a)

continuity constraints (12b)

$$\phi_{k}^{\min}\leq \phi_{k}\left(w_{k}\right)\leq \phi_{k}^{\max}$$ (12c)

where the optimization variables **w**_*k*_, the system dynamics and the additional constraints depend on the chosen transcription and the problem specifics. The NLP is then solved according to a desired technique among the wide range of numerical approaches. This section presents the two transcriptions methods implemented in the Horizon library.

#### 3.4.1 Multiple Shooting

**FIGURE 2:**
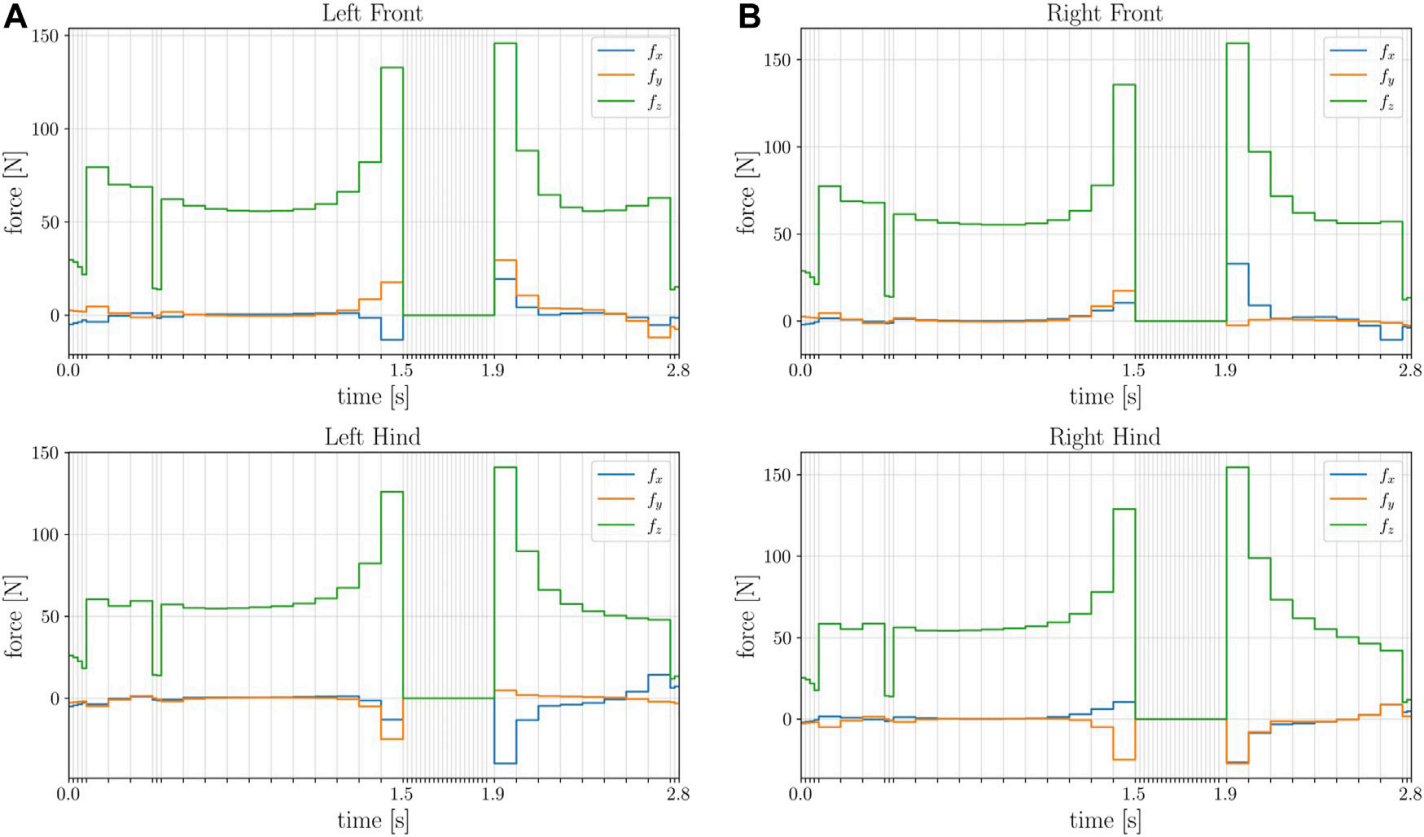
Time history of the contact forces at each foot of the robot. Notice how the forces, being a control input, are constant over each interval.

The multiple shooting method satisfies the constraints (12a) and (12b) by integrating the dynamics of the system over each interval [*t*_*k*_, *t*_*k*+1_] simultaneously, and solving an Initial Value Problem (IVP) to propagate the trajectory between nodes, given the next state **x**_*k*+1_ and the current input **u**_*k*_, as depicted in Figure 2A. Hence, shooting independent trajectories from each initial point and setting a continuity constraint for each pair. The optimization variables **w**_*k*_ involved in (Eq. 11) are the state and the input of the system at each node *k* ∈ [0, *…* , *N*]:

$$w_{k}=\left[x_{k};u_{k}\right]$$ (13)

The ODE solution is computed numerically on each interval [*t*_*k*_, *t*_*k*+1_] given the system dynamics (8).

$$x_{k+1}=x_{k}+\int_{t_{k}}^{t_{k+1}}f\left(x;u;p\right)dt$$ (14)

At the price of increasing the size of the problem (more variables corresponding to each shooting segment, hence more constraint and cost function), the solution is more stable. The fragmentation of the trajectory causes also the drawback of the initialization: relying on many starting points, multiple shooting methods require a good initial guess for each one, otherwise they suffer from poor conditioning. Jacobian sparsity is a direct consequence of this formulation: variables in one node only affect constraints close to that node. These Jacobian forms can be consumed by highly efficient state-of-the-art solvers to accelerate the optimization.

#### 3.4.2 Direct Collocation

Direct collocation approximates state and control using piece-wise continuous polynomials. The integration to simulate the evolution of the system between nodes is converted to an algebraic equation dependent on a few parameters. Doing so, the computation time of the integration is ruled out. To shape the polynomial to follow the system dynamics, an auxiliary set of constraint is set to impose dynamics on intermediate points, called *collocation points*.

Sparsity is guaranteed by the formulation of the transcription: the robot dynamics must be satisfied solely at the collocation points, resulting in a sparse Jacobian matrix that can be handled by state-of-the-art NLP solvers. The direct collocation method, similarly to multiple shooting, discretize the trajectory as in (Eq. 10), and, as a result, the control inputs and the state variables. Moreover, it adds to each interval [*t*_*k*_, *t*_*k*+1_] a set of *d* supplementary nodes: the trajectory is approximated as a Lagrange polynomial parametrized through the *d* way-points, called *collocation points*, as shown in Figure 2B. A Lagrange polynomial has the advantage that its value at each collocation point is exactly the value of the collocation point. Hence, the optimization variables **w**_*k*_ involved in (Eq. 11) are the state **x**_*k*_ and the input **u**_*k*_ at each node *k* ∈ [0, *…* , *N*], plus the states **x**_*c*_ at every collocation node *c* ∈ [1, *…* , *d*] for each interval:

$$w_{k}=\left[x_{k};x_{c}^{1};\ldots ;x_{c}^{d};u_{k}\right]$$ (15)

The direct collocation method satisfies the constraints (12b) by guaranteeing continuity over the whole trajectory, while the constraint (12a) is imposed by evaluating the time derivative of the collocation polynomial at all collocation points and constraining it to the dynamic of the system at that way-point:

$$\begin{array}{cc} p_{c}\left(t_{k+1}\right) & =x_{n+1}\\ {\dot{x}}_{c} & =f\left(x_{c},u_{c}\right) \end{array}$$ (16)

where *t*_*c*_ can be normalized at each interval. The term **x**_*c*_ and **u**_*c*_ corresponds to the state trajectory evaluated at time *t*_*c*_. Notice that the input **u** is piece-wise constant over each interval [*t*_*k*_, *t*_*k*+1_]. By forcing the derivative of the polynomial at each collocation point to be equal to the system dynamics, the resulting function in each interval approximates the real system behaviour, rather than integrating an initial state to compute the exact dynamics as in Section 3.4.1. Indeed, inserting additional collocation points reduce the approximation error and at the cost of increasing the dimension of the problem: each new collocation point amounts to add a new variable and the corresponding dynamic constraint.

## 4 Framework Description

**FIGURE 3:**
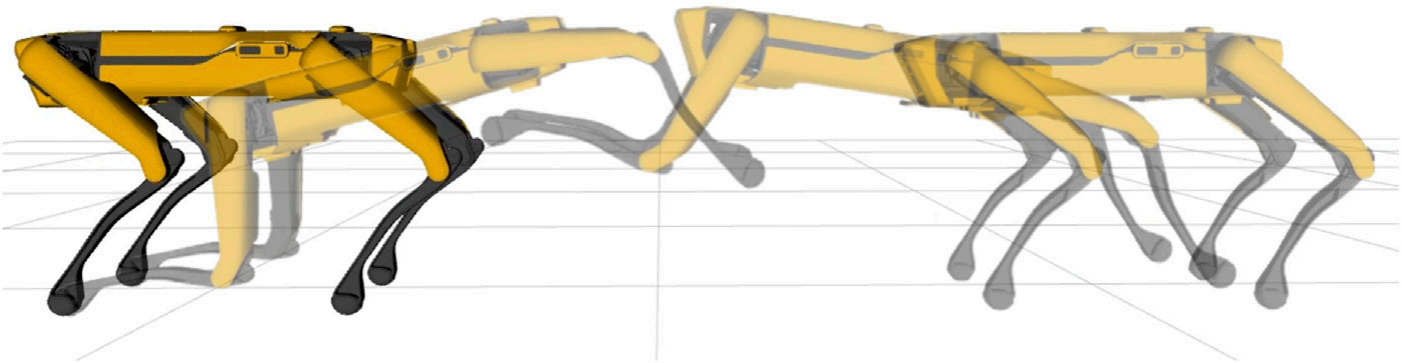
Frame sequence of Spot^®^ while performing a leaping motion. The quadruped robot jumps using the hind legs to provide thrust, and finally lands on the front legs at a specified position.

Horizon is a direct optimal control open-source toolbox tailored to robotics that assembles the steps required for efficient trajectory optimization in a flexible and modular pipeline. It is conceived as a ready-to-use tool that simplifies the prototyping of robot motions. In fact, it offers the possibility to be used as a self-contained black-box tool, divided into independent modules. Namely, robot model parsing, problem definition and NLP transcription, state-of-the-art solver interfaces and trajectory manipulation for a correct deployment on the robot. Its module-oriented design facilitate the coupling with existing frameworks and allows for customization, as each module is extendable with user-written routines. This section provides a detailed description of each module and how they combine in a organic framework capable of designing highly dynamic manoeuvres and, more in general, optimal trajectories for any robotic platform. A schematic representation of Horizon’s main components and their interaction is given in Figure 3.

### 4.1 NLP transcription

Among the spectrum of viable ways to define an algorithm that can be solved numerically, most of the optimization problems in the category of dynamic motion planning are solved using either direct collocation or multiple shooting. Horizon implements these two simultaneous methods: this implies that input and state trajectories are parametrized as decision variables over a number of intervals, and the system dynamics are enforced through the chosen method, either multiple shooting or direct collocation. The modular scheme of Horizon allows the user to use a custom transcription instead of relying on the default ones. Multiple shooting constraints uses a default integrator (RK-4), but it can be easily switched to any other integrator.

### 4.2 Integrators

Horizon includes a collection of numerical integrators, offering a trade-off between simulation speed and accuracy. The possibility to select a desired integrator is useful, since the solution is highly dependent on the integrator details, as discussed in Appendix.

In particular, Horizon provides the following integrators implementations, all supporting both fixed and variable time:

• *Forward Euler (FE)*: this method is based on a truncated Taylor series expansion. An error is induced at every time-step due to the truncation of the Taylor series, this is referred to as the Local Truncation Error (LTE) of the method. For the forward Euler method, the LTE is O(d*t* ^2^). Hence, the method is referred to as a first order technique.
• *Runge-Kutta*: the FE method has rather poor convergence to the solution, the rate of convergence scaling linearly with d*t*. The Runge-Kutta methods are a class of methods which judiciously uses the information on the slope at more than one point to extrapolate the solution to the future time step. The classical second order accurate Runge-Kutta method (RK2), where the LTE is O(d*t* ^3^). In a similar fashion Runge-Kutta methods of higher order can be developed. One of the most widely used methods is the fourth order Runge-Kutta (RK4) technique. The LTE of this method is O(d*t* ^5^).
• *Leap-Frog*: a time-reversible second order accurate method that guarantees conservation of energy. See Appendix for a detailed description of the method.

### 4.3 Node Duration Optimization

The classical approach to the discretization of the system partitions the time domain into a fixed grid. However, in many applications it would be ideal to let the solver decide the duration of some portion of the trajectory: for instance, if the robot is performing a jumping motion, the flight time should be flexible. Another example is any motion for which it is not known a priori the total duration. Yet another example is contact timing in a desired gait, which can be optimized jointly with contact location and contact forces. An additional reason to adopt a variable d*t* is to reduce the number of shooting nodes, by letting the solver to choose the discretization time. In fact, under some circumstances (usually quasi-static motions), a large d*t* does not generate dynamically inconsistent outcomes, while in other cases (agile and fast actions), a small d*t* is essential. Most of the dynamic motions planned are a mix of the two scenarios: while the number of nodes keeps constant, if the sample times are made themselves decision variables, the solver is able to stretch or shrink the time intervals between the nodes. However, the flexibility of the time grid comes with a the considerable drawback of an increased number of decision variables, that could lead to higher computation times or bad conditioning of the problem.

### 4.4 NLP Solvers

The NLP formulation of a motion planning problem with complicated dynamics produces hard, non-convex problem, due to the combination of geometric (e.g. environment constraints), kinematic (position, velocity and acceleration bounds) and dynamic constraints. However, thanks to the uncoupled formulation of simultaneous methods, changes in the inputs only affects the state at a given node, while future states are unaffected. This leads to a structure of the problem that is inherently sparse, making it suitable for large-scale nonlinear solver such as IPOPT. According to the authors’ experience, and given the wide variety of NLP problems that come out of an Optimal Control Problem (OCP), it is very likely that a one-size-fits-all solver does not exist. On the contrary, a specific solver can be more prone to finding a suitable solution to the task at hand. For this reason, Horizon makes a number of different solvers available to the user, and allows to easily switch between them. More specifically, Horizon includes the following NLP solvers:

1) A custom implementation of a sparse, Gauss-Newton Sequential Quadratic Programming (GN-SQP) algorithm;
2) A custom implementation of a multiple-shooting Iterative Linear-Quadratic Regulator (ILQR);
3) IPOPT, and state-of-the-art solvers, that are made available by CasADi’s nlpsol interface.

In particular, IPOPT is a large scale nonlinear solver, based on a primal-dual interior point method. This state-of-the-art solver is used off-the-shelf, as it is known to perform adequately on a wide range of problems. The choice to implement two other custom solvers is aimed at covering some structural limitations of interior-point methods, and also with the purpose to exploit the specific structure of an OCP. Table 2 highlights the differences with reference to a few relevant properties, such as 1) the ability to exploit a good initial guess through warm-starting, 2) linear computational complexity w.r.t. the horizon length, and 3) the ability to deal with inequality constraints (bounds).

**TABLE 2:** Comparison between solvers provided by the Horizon package.

Solver	Warm-Starting	Guaranteed *O*(*N*)	Bound Support
IPOPT	No	No	Yes
GN-SQP	Yes	No	Yes
ILQR	Yes	Yes	No

#### 4.4.1 GN-SQP

Sequential Quadratic Programming (SQP) is a well-known and effective method for non-linear constrained optimization. It consists in generating steps by solving quadratic sub-problems. SQP methods show their strength when solving problems with significant non-linearities in the constraints.

Given a generic NLP in the form:

$$\begin{array}{cc} \underset{x}{\min} & \;\ell \left(x\right)\\ \text{ subject to } & \;c\left(x\right)=0,\\ & \;b\left(x\right)\geq 0, \end{array}$$ (17)

and its Lagrangian:

$$L\left(x,\lambda ,\mu \right)=\ell \left(x\right)-\lambda^{T}c\left(x\right)-\mu^{T}b\left(x\right),$$ (18)

the SQP method computes a new iteration for the Newton step, namely *δ* **x**_*k*_, by solving a local QP sub-problem in the form:

$$\begin{array}{cc} \underset{\delta x_{k}}{\min} & \;\ell \left(x_{k}\right)+\nabla \ell {\left(x_{k}\right)}^{T}\delta x_{k}+\frac{1}{2}\delta x_{k}^{T}\nabla_{x,x}^{2}L\left(x_{k},\lambda_{k},\mu_{k}\right)\delta x_{k}\\ \text{ subject to } & \;\nabla c{\left(x_{k}\right)}^{T}\delta x_{k}+c\left(x_{k}\right)=0,\\ & \;\nabla b{\left(x_{k}\right)}^{T}\delta x_{k}+b\left(x_{k}\right)\geq 0. \end{array}$$ (19)

In the particular case of a NLP with a least-squares objective function, i.e.

$$\ell \left(x\right)=\frac{1}{2}{\left\|h\left(x\right)\right\|}_{2}^{2}$$ (20)

it is convenient to adopt the so-called *Gauss-Newton (GN)* approximation of the Hessian of the Lagrangian:

$$\nabla_{x,x}^{2}L\left(x_{k},\lambda_{k},\mu_{k}\right)=J{\left(x_{k}\right)}^{T}J\left(x_{k}\right),$$ (21)

with $J\left(x\right)=\frac{\partial h\left(x\right)}{\partial x}$ . The GN approximation has the advantage of always leading to a convex sub-problem; it also does not depend explicitly upon the Lagrangian multipliers, and is cheaper to compute w.r.t. the full Hessian.

Horizon implements a sparse GN-SQP method which is written in C++, and exploits Eigen3 and CasADi for linear algebra and derivative computation. On the back-end, the OSQP solver is employed for the solution of each sub-problem in (Eq. 19). The implemented globalization strategy is based on a line-search procedure acting on a suitable merit function.

#### 4.4.2 Iterative LQR

Modern libraries for sparse linear system solving (such as those provided by the *HSL* and *SuiteSparse* projects, and others) typically manage to exploit the structure of a generic NLP program, so that its complexity is heuristically found to be way lower than the *O*(*N* ^3^) baseline. Optimal control problems, however, are known to exhibit a peculiar structure due to their *Markovian* nature, and this reflects to a specific sparsity pattern that can be exploited by a tailored solver, known as the Iterative Linear-Quadratic Regulator (ILQR)^4^ solvers. The core of such a family of solvers is the well-known Riccati backward recursion, which exploits the Bellman’s principle of optimality to reduce the NLP single-iteration complexity from *O*(*N* ^3^), which corresponds to a dense algebra implementation to *O*(*N*), where *N* is the horizon length. The Horizon package provides a custom implementation of ILQR that is fully integrated into Horizon’s API. It is written in C++ for efficiency, and exploits CasADi for automatic differentiation (including C code generation of derivatives), and the well-known Eigen3 library for linear algebra.

We base our implementation upon the work of Giftthaler et al. (2018b) in order to support both single-shooting and multiple-shooting formulation. Differently from other available packages, our implementation supports exact equality constraints, both in the form of state-input constraints, as well as pure state constraints, while still enjoying linear complexity in the horizon’s length. Globalization strategies are based on either a filter method, or a classical merit-based line search (see Wächter and Biegler (2005); Nocedal and Wright, (2006)), depending on the user’s choice.

### 4.5 Mesh Refinement

The solution vector is a discrete trajectory defined at each node, that, in turns, corresponds to a fixed time instant. Depending on the formulation of the problem, the nodes can be uniformly distributed, if the step size d*t* is constant, or positioned unevenly if d*t* is set as an independent optimization variable. In both scenarios, a common requirement is the manipulation of the optimal solution in order to retrieve a post-processed trajectory with a sampling rate that is 1) constant and 2) adequate to represent the frequency content of the solution.

**FIGURE 4:**
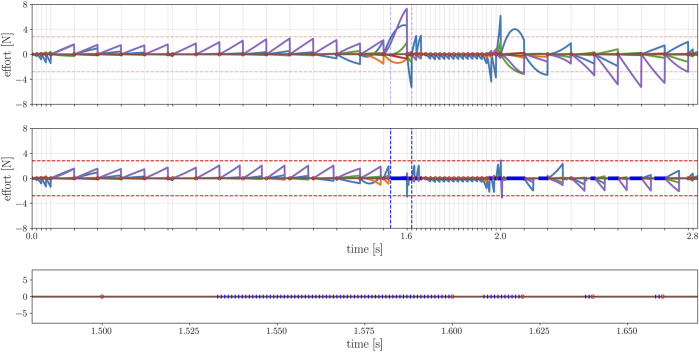
Effort on the floating base. Above: re-sampling the original trajectory (which satisfies the dynamic constraint at each node) at a higher frequency (1 KHz) produces high efforts (colored lines) on the virtual joints of the robot, due to the divergence in between nodes. Bottom: the mesh-refinement restores up to the threshold (red horizontal lines) the dynamic feasibility, reducing the efforts on the floating base. The red dots represent the nodes of the original problem, whereas the blue segments are the new nodes injected by the refiner. The blue vertical lines highlight a portion of the trajectory where supplementary nodes were injected, detailed in the last plot.

The number of nodes corresponds to a given frequency in the time domain of the optimal solution. However, between the optimal solution found and the trajectory sent to the robot there is a layer of re-sampling: as a matter of fact, in a trajectory optimization problem should be used the least possible number of nodes to reduce the problem complexity, while the reference trajectory sent to the robot is usually sampled at high frequencies. If the *direct collocation* strategy is used, re-sampling the solution is trivial, since both the control and the state are exact polynomials that can be re-sampled to any frequency. On the other hand, if the multiple shooting strategy is opted for, no analytic description of the trajectory is given. A naive approach, such as polynomial interpolation, may not follow the dynamics of the system in between the nodes. Thus, a different approach is preferred: at each interval [*n*_*i*_, *n*_*i*+1_], the same ODE integrator used to transcribe the problem is employed to integrate the system with initial value **x**_*i*_ subjected to the constant input **u**_*i*_. This procedure yields a re-sampled trajectory at a given frequency that meets the system dynamics condition. However, due to the time gridding imposed by the NLP transcription, the constraint functions act only on the nodes (i.e. at certain time instants), while the interval between each couple of node is unconstrained: the optimal solution is only feasible on the nodes, and extracting new samples by integrating the previous state with a constant input may lead to constraint violations along the trajectory. This may render the re-sampled solution unfeasible: for example, by introducing parasite torques on the floating base, as shown in Figure 4.

**FIGURE 5:**
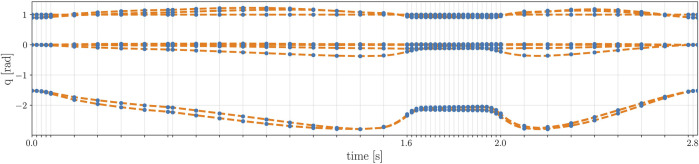
Optimal joints trajectory of the robot before and after the mesh refinement. The optimal solution of the original problem, in blue, coincides with the re-sampled trajectory after the mesh refinement, in orange. As it can be noticed, the samples of the original trajectory have a variable time step, while the re-sampled one has a constant frequency of 1 KHz.

Horizon offers a tool to reduce these re-sampling errors and without considerably changing the original optimal trajectory, as shown in Figure 5.

Algorithm 1 Mesh refinement.

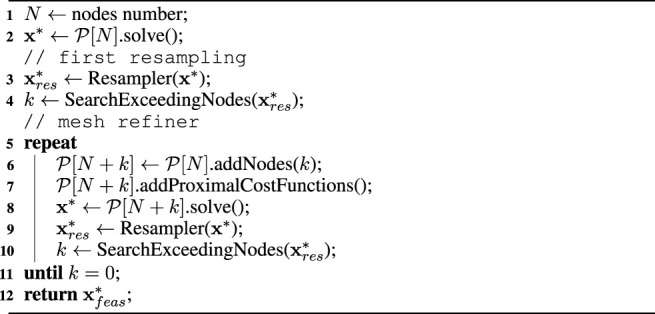

The aim of the mesh refiner is to reduce re-sampling error by decrease the grid spacing of the discretization to avoid constraint violations. This could be achieved by increasing the number of nodes over the whole time horizon, but it would lead to a very high number of variables and constraints and make the problem intractable. The mesh refiner, on the contrary, acts locally by adding nodes only where it is required. The pseudo-code is given in Algorithm 1: first, it searches for the *k* values of the re-sampled trajectory $x_{\mathrm{res}}^{*}$ that exceed a desired feasibility threshold. Then, it injects *k* supplementary nodes in the original problem P[N] in order to locally increase the node density. The resulting problem has *N* + *k* nodes. Indeed, all the variables, the constraint and the cost functions specified in the original problem must be updated according to the new discretization of the time horizon. The feasibility threshold is relative to the optimal solution, which depends on the formulation of the problem: in the case of a floating-base robot, the first violation to check is the dynamic feasibility, as the torque of the virtual joints should indeed be zero. Differently, the violations induced by the re-sampling in a fixed-base robot may involve torque or acceleration limits imposed by the hardware specification. More generally, the feasibility threshold depends on the type of the optimization problem at hands. For instance, the graph in Figure 4 refers to the violation of the dynamic feasibility constraint in the case study presented in Section 5. The aim of the new optimization problem is not to find a novel solution, but to search for the solution closest to the original one that satisfies the constraints over a finer time grid. Hence, some simplifications can be made to the problem formulation to reduce the problem complexity and start the optimization as close to the desired solution as possible:

• if the step size d*t* was specified as an optimization variable, it is converted to a parameter using the previous d*t* values.
• P[N+k] is initialized with the optimal solution values of the original problem P[N] : on the *N* nodes coinciding with P[N] , each variable’s initial guess is set to the corresponding value in the optimal solution **x**∗. Similarly, the *k* injected nodes in between the original *N* nodes are initialized with the values of the re-sampled trajectory $x_{\mathrm{res}}^{*}$ .
• suitable bounds are assigned to variables and constraint functions on the *k* injected nodes: in particular, for the *j*th injected node in [*n*_*i*_, *n*_*i*+1_], each bound at *n*_*j*_ is set to the corresponding value at *n*_*i*_.

Finally, a set of auxiliary cost functions is set, i.e. the *proximal cost functions*: these terms are required to steer the optimization towards the original solution. For instance, the state proximal cost functions ‖**x**_*res*_ − **x**‖^2^ or the input minimization ‖**u**_*res*_‖^2^. Depending on the nature of the problem, custom proximal terms can be added to the mesh refiner. The mesh refining process is repeated until a feasible solution $x_{\mathrm{feas}}^{*}$ is found.

### 4.6 Utilities

Horizon comes with a collection of secondary utilities to simplify the problem construction and analysis. In the context of motion generation and prototyping, a tool to save and load the optimized trajectory can be useful. The Horizon *MatStorer* is in charge of managing the obtained solution, saving it along with the relevant parameters of the problem. This allows to up-cycle the solution to warm-up a similar problem. The code below shows how to use such a tool.


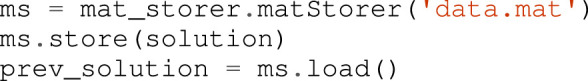


Furthermore, it is useful for analysis and inspection if coupled with the Horizon *Plotter* and *Replayer* modules. The first tool streamlines data plotting of state and input variables, constraints and the relative bounds, while the second relies on the ROS tool RViz^5^ to visualize the obtained trajectory. A code snippet that achieves the desired behavior is the following:


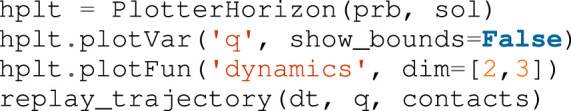


Finally, Horizon makes available a re-sampling tool that evaluates a trajectory at a desired frequency, which is a necessary step towards robot deployment. This is especially useful when dealing with problems that also optimize over the time step *dt*, as described in Section 4.3. Since the optimal solution of such problems has an irregular time discretization, a re-sampling layer is required to obtain a trajectory with a fixed frequency. The code below shows a simple usage of the re-sampling module:


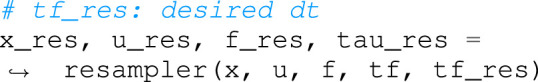


### 4.7 Model Acquisition

The first module of Horizon is devoted to the parsing of the robot model in a form that is compatible to the underlying symbolic framework. It leverages Pinocchio, which provides the robot kinematics and dynamics exploiting state-of-the-art Rigid Body Algorithms for articulated systems. The parser module guarantees ease-of-use and compatibility, as it supports the Universal Robot Description Format (URDF)^6^, commonly used for the description of the robot model. Given the URDF file as input, it exposes useful algorithms related to kinematics and dynamics, such as the *Articulated Body Algorithm (ABA)* and the *Recursive Newton-Euler Algorithm (RNEA)* (Featherstone, 2014), as well as forward kinematics, computation of Jacobians, and others.

The dynamics and the analytical derivatives of the model discussed in Section 3.1 are provided by the parser in a symbolic form, so that they can be injected into the optimization: throughout the problem definition, kinematics and dynamics information can be retrieved as symbolic variables that can be used in constraints and objective functions. This is achieved through the full scalar templatization of the Pinocchio library: the Horizon parser implements it using the types of CasADi, that, as presented in Section 3.3, is the backbone of the framework’s symbolic layer. The following lines of code shows how to initialize the model from the URDF file:


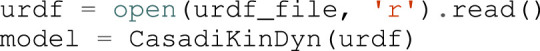


### 4.8 Problem Formulation

As described in 3.2, the trajectory optimization problem is transcribed into an NLP problem with a fixed number of nodes and intervals. As a result, each function is parametrized as a vector of decision variables, one for each node of the trajectory. This is true for state and input vectors, constraints and cost functions. To provide an intuitive interface, the design choice of Horizon is to keep separated the notion of optimization variable from its implementation at each node: the user is simply returned a handle to the *abstract variable*, which can be used to define generic constraint and cost functions and select the nodes on which they are active. An underlying layer is in charge of expanding each variable, constraint and cost function over the corresponding nodes. Indeed, if necessary, the user can easily access the value at each node. Horizon provides several methods to structure the problem by adding different types of constraints, such as control and state bounds, intermediate and terminal constraints. Additionally, being a toolkit specifically tailored to robotics, Horizon implements floating-base constraints, such as under-actuation and friction cones, as well as a set of geometric constraints to build environment obstacles and intuitive methods to constrain end-effector position, velocity and acceleration. The set list to generate a minimal working problem reads as follows.

1. *Trajectory discretization*: define a number of intervals and set it to the optimization problem. The optimization horizon is divided into *N* segments. The variables and functions defined later in the problem will be parametrized accordingly: the state trajectory is divided into *N* + 1 nodes to include the final state, while the input is defined as a piece-wise constant function over the *N* intervals. The optimal problem is then initialized as follow:

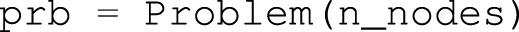

2. *Variables definition*: create the decision variables of the optimization problem and specify their properties along the trajectory, such as their bounds and initial guess at each node. Horizon offers the three types of possible definitions:
  • independent variable: the abstract variable is expanded as a vector of independent variables on each node. Some relevant methods are shown in the code snippet below:

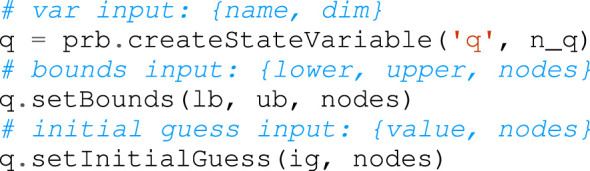

• single variable: only one decision variable enters the optimization problem, with the same value on each node. The code to instantiate it is the following:

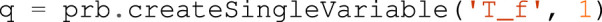

3. *Parameter definition*: create a parameter with the same syntax of a variable. A parameter is a symbolic variable that does not enter the optimization problem and can be set to a desired value at any time, even after the building of the problem. This is particularly useful when imposing reference trajectories in a receding horizon formulation, as they change at each iteration. The syntax to create a parameter is similar to the above-mentioned variables:

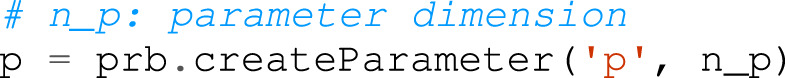

4. *Dynamics injection*: the problem requires the dynamics of the system, formulated as differential-algebraic system of equation (DAE). Inserting the dynamics and the step size (which can be fixed or an optimization variable) is mandatory:

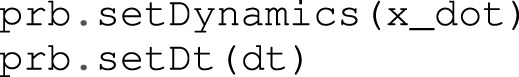

5. *Function definition*: assemble functions as constraints or costs. Similarly to a decision variables, each function can be added to a desired set of nodes. The constraints can be bounded. Each cost function is a component of the objective function to be minimized. The relevant methods to create constraint and cost functions is shown below. Notice how it is possible to define a constraint function (and its bounds) over a portion of the whole trajectory by specifying the desired nodes:

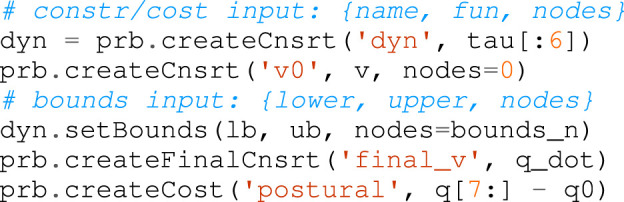

6. *Problem transcription*: select the transcription method that will be added as a set of constraints along the trajectory. Two methods are available: direct collocation and multiple shooting. As introduced in Section 3.4, the multiple shooting method requires an integrator that can be selected from those listed in Subsection 4.2 or manually implemented:

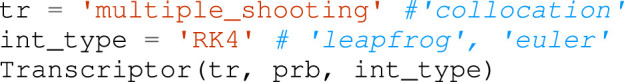

7. *Problem building and solving*: Horizon sets up the problem. It expands the variables on each node and generate the corresponding derivative matrices for the solver selected. A variety of solvers can be chosen to solve the problem. Horizon exposes all the solvers supported by CasADi and provides two custom solvers, ILQR and GN-SQP:

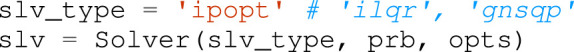


Indeed, the optimized trajectory is a combined result of the formulation of bounds, constraints and cost functions: the desired behaviour can be steered by the formulation of the problem. The performance index is a measure of the “quality” of the trajectory. A high (or low) value of the performance index corresponds to the closeness of the trajectory to the desired behaviour. Refining the resulting motion is a part of trajectory prototyping that can be tackled by adding new elements to the cost function, tune the weights of each component of the objective function or provide the solver with an initial guess that is close to the expected local minimum.

## 5 Case Study

This section provides an in-depth exploration of the capabilities of the framework by presenting a case study. The goal of this case study is to produce a vertical jump with a 120 deg twist using the robot Spot^®^.

### 5.1 Problem Formulation

Given a URDF description of the robot Spot^®^, the model is imported in a symbolic form. Horizon returns an handle to model instance, which is used throughout the problem. Spot^®^ is a four-legged robot, for a total of *n*_*j*_ = 12 degree of freedom (DoF) distributed evenly on the four limbs. Each leg is equipped with three actuators, granting two DoF at the level of the hip (roll and pitch) and one at the knee level. The robot has *n*_*c*_ = 4 contact points, corresponding to the tip of each leg $c=\left[c_{lf},c_{rf},c_{lh},c_{rh}\right]$ . The initial step is choosing a suitable number of nodes to discretize the time window. This choice implies a trade-off between the resolution of the time grid and the dimension of the problem. For this particular problem, we split it into 50 portions, which corresponds to *N* = 51 nodes. As introduced in 3.2, the state of the system is divided into *N* decision variables, one for each node, while the input is defined for the *N* − 1 edges connecting the nodes. The aim of the optimization is to find a feasible motion that corresponds to the robot jumping vertically: the optimization ought to find joint acceleration and torques that satisfy this manoeuvre, as well as the forces exerted at each end-effector. Among the many possible design choices, we opt for the formulation in (Eq. 4), where $x\in R^{n_{q}+n_{v}}$ is the state vector of joint position and velocity combined, whereas the input is chosen as:

$$u=\left[\begin{array}{c} \dot{\nu }\\ f_{c}\\ \text{d}t \end{array}\right]$$ (22)

where $\dot{\nu }\in R^{n_{v}}$ represents the generalized accelerations, $f_{c}\in R^{3\cdot n_{c}}$ are the Cartesian forces at each contact and dt∈R is the time step between each node.

Specifying the ***dt*** of the problem as a variable is particularly useful when there are phases of the motion that needs to be stretched or shrunk to guarantee feasibility. A jumping motion is imposed framing it between one take-off and one landing node that are fixed. If the ***dt*** is kept constant, choosing a fixed number of nodes also fix the duration of the jumping motion, constraining in turn the forces and the overall motion of the robot, hence reducing the freedom of the system. By selecting the ***dt*** as an independent variable, the optimizer can adapt the time length of the flight phase, for instance, on the height of the jump or the maximum torque available. The solver will choose a suitable duration of each node so as to output a feasible trajectory. In fact, the subsequent phase consists of adding lower and upper bounds to guarantee plausible solutions (e.g. acceleration or torques) that can be deployed on real hardware. For each state and input variable lower and upper bounds are added on each node. Furthermore, on the first node the joint position is set to the initial robot configuration while the velocity is bound to zero:

$$\begin{array}{cc} q^{0}=q_{\mathrm{init}}\; & \text{initial position}\\ \nu^{0}=0\; & \text{initial velocity}\\ q_{min}^{k}\leq q^{k}\leq q_{max}^{k}\; & \text{position bounds}\;\forall k\in \left[1,N-1\right]\\ \nu_{min}^{k}\leq \nu^{k}\leq \nu_{max}^{k}\; & \text{velocity bounds}\;\forall k\in \left[1,N-1\right]\\ {\dot{\nu }}_{min}^{k}\leq {\dot{\nu }}^{k}\leq {\dot{\nu }}_{max}^{k}\; & \text{acceleration bounds}\;\forall k\in \left[1,N-1\right]\\ f_{c,min}^{k}\leq f_{c}^{k}\leq f_{c,max}^{k}\; & \text{contact force bounds}\;\forall k\in \left[1,N-1\right] \end{array}$$ (23)

Additionally, an interval for the variable ***dt*** is fixed, in order to avoid trivial solutions that could collapse the trajectory.

$${\text{d}t}_{\min}^{k}\leq {\text{d}t}^{k}\leq {\text{d}t}_{\max}^{k}\;\forall k\in \left[1,N\right]$$ (24)

When prototyping a new robot motion, the initial guesses cannot be easily provided to the solver, unless a library of primitives is available. However, as soon as a rudimental solution is found, it can be used as a valuable initial guess to refine the same trajectory or a family of similar motions (see Section 4.5). The following step is selecting a transcription method. Horizon offers two built-in strategies, multiple shooting and direct collocation, presented in Section 3.4. For this problem, we decide upon the multiple shooting method using the default Runge-Kutta integrator. Once specified, Horizon formalize it by creating the integrator and a set of constraints on each node to guarantee continuity.

The next phase consists of imposing the necessary constraints to achieve a desired result. The first constraint impose the under-actuation of the system, as Spot^®^ is a floating base robot with four contacts on the real and the front legs. The equations of motion are provided by the model instanced by the Horizon parser using the RNEA algorithm. Given the vector of Cartesian forces at each contact $f_{c}=\left[f_{c}^{lf},f_{c}^{rf},f_{c}^{lh},f_{c}^{rh}\right]$ , a symbolic representation of the inverse dynamics torques $\tau \in R^{n_{v}}$ can be retrieved from the general form of (Eq. 3), which we report here for convenience:

$$\tau =M\left(q\right)\dot{\nu }+h\left(q,\nu \right)-J_{c}^{⊤}f_{c}.$$ (25)

Note that in (Eq. 25) the vector ***τ*** represents the efforts acting on all the degrees of freedom of the robot, including the floating base. Therefore, to enforce under-actuation, the first six elements of such a torque vector are constrained to zero, whereas the joint torques ***τ***_*j*_ are bound inside the motor limits on every node:

$$\tau_{fb}^{k}=0\;\forall k\in \left[0,N\right]$$ (26)

$$\tau_{jmin}^{k}\leq \tau_{j}^{k}\leq \tau_{jmax}^{k}\;\forall k\in \left[0,N\right]$$ (27)

### 5.2 Shaping the Robot Motion

Up until now, the structure of the optimization problem for a quadruped robot is very generic. This section present how Horizon can be used to prototype a jumping motion in a simple and intuitive way. The first constraint imposes a terminal velocity: at the end of the motion, the robot should be standing still. This rule amounts to add a single constraint at the last node forcing the joint velocity to be zero:

$$\nu^{N}=0$$ (28)

To impose a jumping behaviour, a portion of the trajectory is designated for the desired action: a take-off node *N*_*to*_ = 20 and a landing node *N*_*la*_ = 40 are selected, corresponding to the *flight phase*. Outside this range of nodes, the Cartesian velocities at each contact ${\dot{p}}_{c}=\left[{\dot{p}}_{c}^{lf},{\dot{p}}_{c}^{rf},{\dot{p}}_{c}^{lh},{\dot{p}}_{c}^{rh}\right]\in R^{3}$ are constrained to be zero:

$$p_{c}^{k}=0\;\forall k\in \left[0,N_{to}\right]\cup \left[N_{la},N\right]$$ (29)

Similarly, at the same nodes the friction cones constraints are imposed: the contact force ***f***_***c***_ is required to lie inside the linearized Coulomb friction cone directed by the surface normal $n_{S}$ :

$$\begin{array}{cc} & f_{c,i}\cdot n_{S}>0,\\ & {\left\|f_{c}^{t}\right\|}_{\infty }\leq \mu_{i}\left(f_{c}\cdot n_{c}\right)\;\forall k\in \left[0,N_{to}\right]\cup \left[N_{la},N\right] \end{array}$$ (30)

which imposes unilaterality and non-slippage of the contact *c*_*i*_. In the above equation, $f_{c,i}^{t}=f_{c,i}-\left(n_{c,i}\cdot f_{c,i}\right)\cdot f_{c,i}$ denotes the tangential component of ***f***_*c*,*i*_, and *μ*_*i*_ the static friction coefficient. On the other hand, inside the *flight phase* the Cartesian forces are constrained to be zero.

$$f_{c}=0\;\forall k\in \left[N_{to},N_{la}\right]$$ (31)

To obtain the twisting motion during the jump, it is enough to constrain the robot postural and the floating base at the last node. Specifically, the orientation of the floating base ***ρ*** is set to the desired one, while the remaining elements, i.e. the floating base ***p*** and the joint ***θ*** positions, are constrained to the initial configuration:

$$\begin{array}{cc} \rho^{N} & =\rho_{\text{des}}\\ p^{N} & =p^{0}\\ \theta^{N} & =\theta^{0} \end{array}$$ (32)

where ***ρ***_des_ is the quaternion corresponding to a rotation of 120 deg. Thanks to (29), the quadruped cannot move the its contacts while touching the ground: therefore, this last condition translates into a jumping motion that orient the robot’s heading at a desired angle while keeping the same joint configuration as the initial one. Given the above-mentioned constraints, the objective function shapes the behaviour of the robot. In particular, two regularization terms are introduced, both for the velocity ***ν*** and the contact forces ***f***_*c*_. The final optimization problem is constructed by Horizon as follows:

$$\begin{array}{cc} \underset{x\left(\cdot \right),u\left(\cdot \right)}{\min} & \overset{N-1}{\underset{k=0}{\sum }}\left(\|\nu^{k}{\|}^{2}+\|f_{c}^{k}{\|}^{2}\right)\\ \text{subject to:}\\ •\; & \text{state and input bounds}\;\left(23\right)\\ •\; & \text{step size bounds}\;\left(24\right)\\ •\; & \text{torque and under}-\text{actuation bounds}\;\left(26\right)\\ •\; & \text{final velocity constraint}\;\left(28\right)\\ •\; & \text{contact consistency constraint}\;\left(29\right)\\ •\; & \text{flight constraints}\;\left(30\right) \end{array}$$ (33)

The solver module relies on a collection of state-of-the-art solvers. For this case study, the large scale non-linear solver IPOPT was selected.

### 5.3 Resampling

The optimal solution is a vector of state values (i.e. the discretized optimal state trajectory) and a vector of input values, constant over each intervals between the node. The frequency of the trajectory depends on the step size ***dt*** of the problem: in this case study, the step size is a decision variable itself. Thus, a resampling layer is required to obtain a trajectory with a desired fixed frequency that can be sent as a reference to the robot. For this problem, we decided to re-sample the trajectory with a frequency of 1 Khz. Once the re-sampled trajectory is computed by the Horizon re-sampler, the module presented in Section 4.5 is devoted to refine the trajectory: as shown in Figure 4, the unfeasible re-sampled trajectory (due to the parasite torques on the floating base) is corrected by locally injecting a set of supplementary nodes that reduce the undesired torques without changing the joint trajectory of the robot, as shown in Figure 5. These results correspond to four iteration of the mesh-refining process described in Algorithm 1.

### 5.4 Results

**FIGURE 6:**
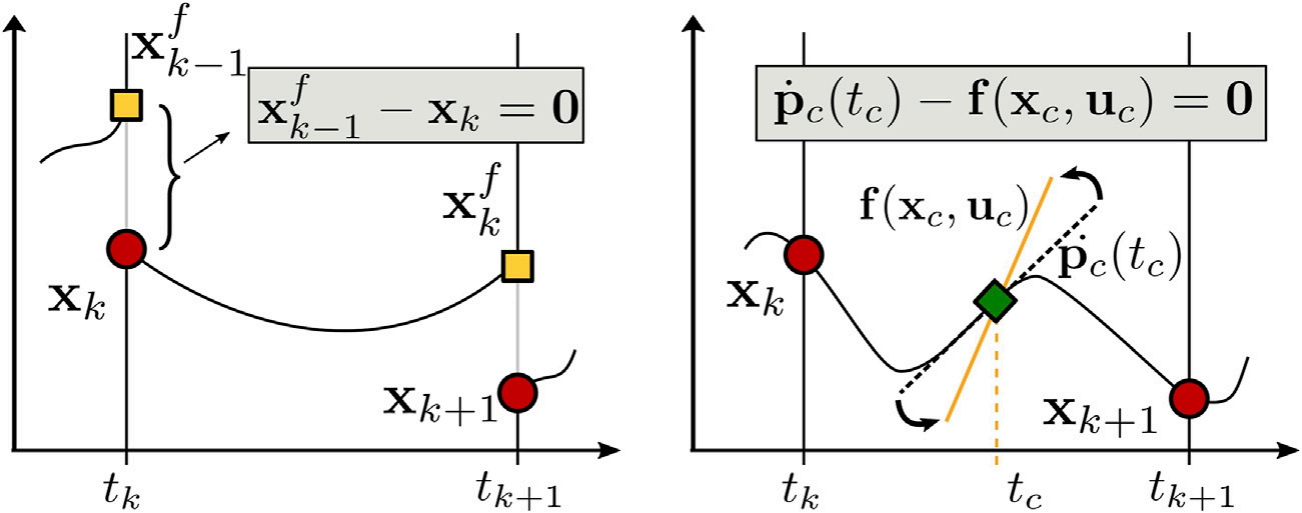
Pictorial comparison between the multiple shooting **(A)** and collocation **(B)** transcription methods. The red dots represent the state vector at each node: the continuity condition in multiple shooting constrains the integrated dynamics (yellow square) over one interval to be equal to the state vector at the next node, while in direct collocation the system dynamics are approximated using a polynomial, setting its derivative (dotted black line) at a given set of intermediate nodes (green square) equal to the system dynamics (yellow line) evaluated at the same points.

**FIGURE 7:**
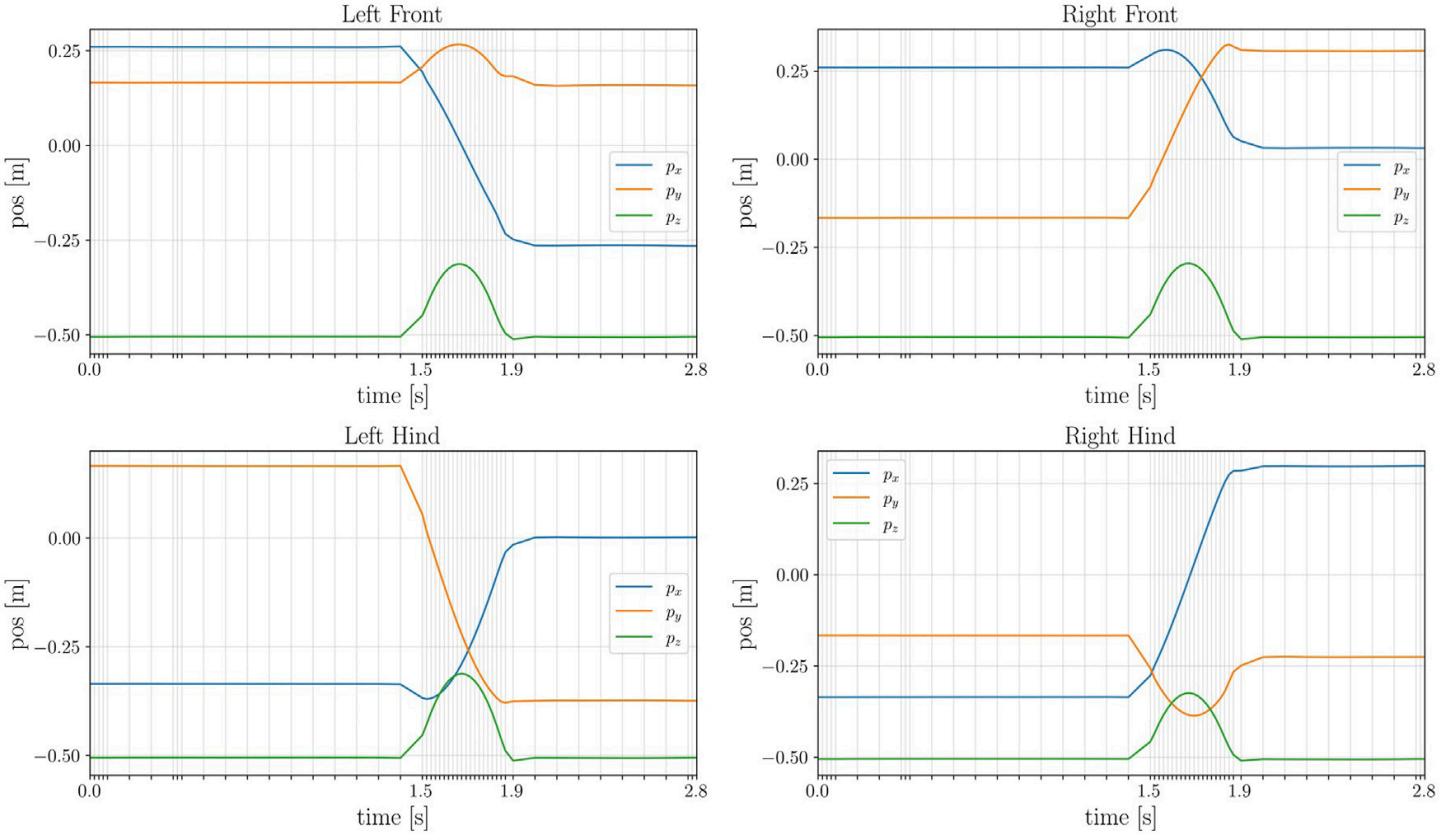
Time history of the contact position at each end-effector of the robot. Notice how the time grid is not evenly spaced: during the jumping motion the optimal step size ***dt*** decreases.

A simple description of the problem produces a complicated and dynamic manoeuvre of the robot Spot^®^: the quadruped successfully jumps while changing its orientation. Figure 6 shows the time histories of the contact forces at each foot, which are simultaneously zero during the flight phase. As it can be noticed, the solver select the optimal step size ***dt*** over the horizon, which decreases during the jump. Figure 7 depicts the trajectory of the feet w.r.t. the world, which is constant over the stance phases. It is worth noticing how the solver exploits the flight phase to change the position of the feet in order to achieve the desired orientation of the robot at the landing. Using a machine equipped with on a AMD Ryzen 7 1700X Eight-Core CPU running at 3.4 GHz, the problem solves in 57 s with 388 iterations. The duration of the final trajectory is 2 s. The selected optimizer is IPOPT, using the default linear solver ma27. As mentioned in Section 4.8, initializing the problem with a adequate guess, the computation time drops considerably. Running the problem a second time using the first result as initial guess, the solver takes only 2.5 s to solve the problem. It is worth noticing that the time elapsed is mainly due to the heavy constrained formulation: if the bounds are relaxed and the constraint reshaped (i.e. the friction cones conditions are removed, the ***dt*** fixed), the time drops to 1.4 s, corresponding to 11 iterations of the solver. It is interesting to notice that the same relaxed problem is solved by GN-SQP in 7.2 s, while ILQR uses 4.2 s. A video of the resulting motion can be found in the accompanying video, which also contains a set of clips to better demonstrate the effectiveness of the framework.

### 5.5 Other Applications

Horizon was validated on a variety of applications involving different robotics platforms. A list containing a brief description of our main achievements is given below.

**FIGURE 8:**
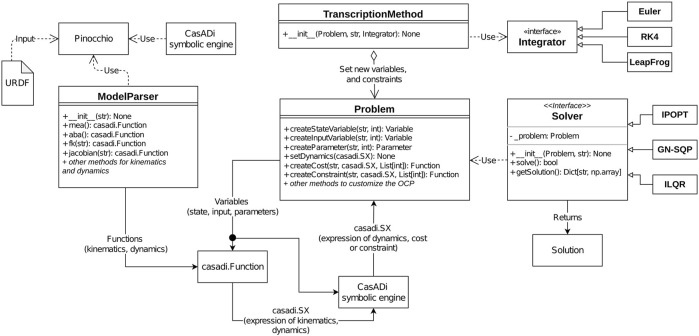
Interaction between the main components of the Horizon framework, as described with a pseudo-UML representation. The core component is the *Problem* component, acting as a manager for optimization variables, parameter, costs and constraints. Variables can be combined thanks to CasADi’s symbolic engine to produce expressions for costs, and constraints. In the robotics domain, dynamics, costs, and constraints can contain expressions related to kinematics and dynamics, as allowed by the *ModelParser* component. A *TranscriptionMethod* component augments the Problem with variables and constraints implementing a transcription strategy. Finally, a *Solver* component translates the abstract representation of Problem into an actual NLP, which is solved to produce a numerical result.

**FIGURE 9:**
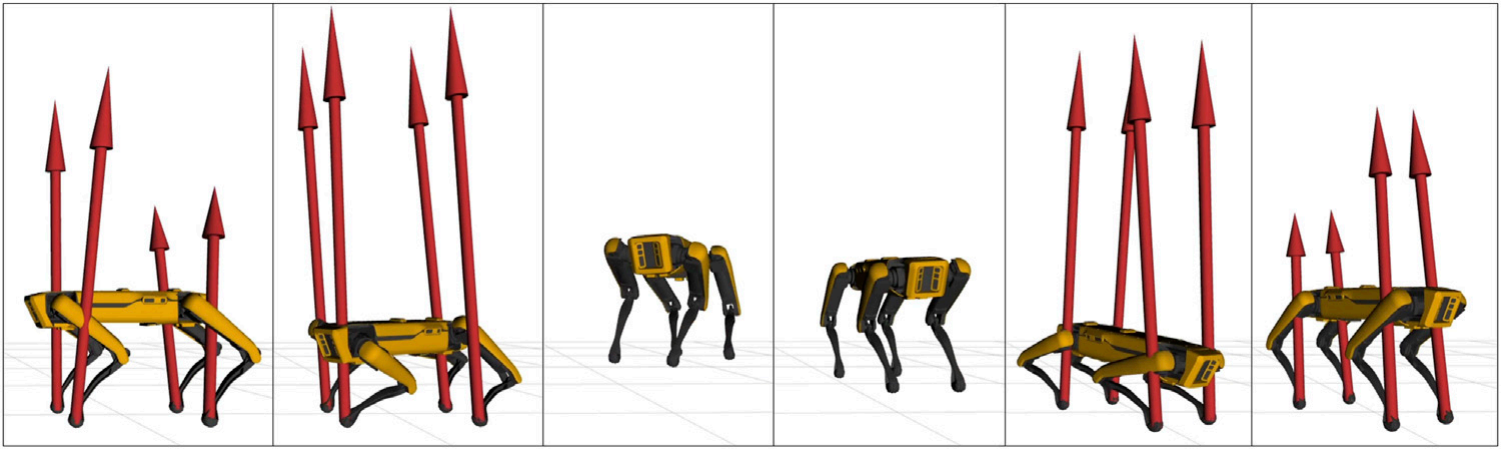
Frame sequence of Spot^®^ jumping vertically while performing a 120 deg twist. Red arrows represent contact forces, constrained to zero during the flight phase.

**FIGURE 10:**
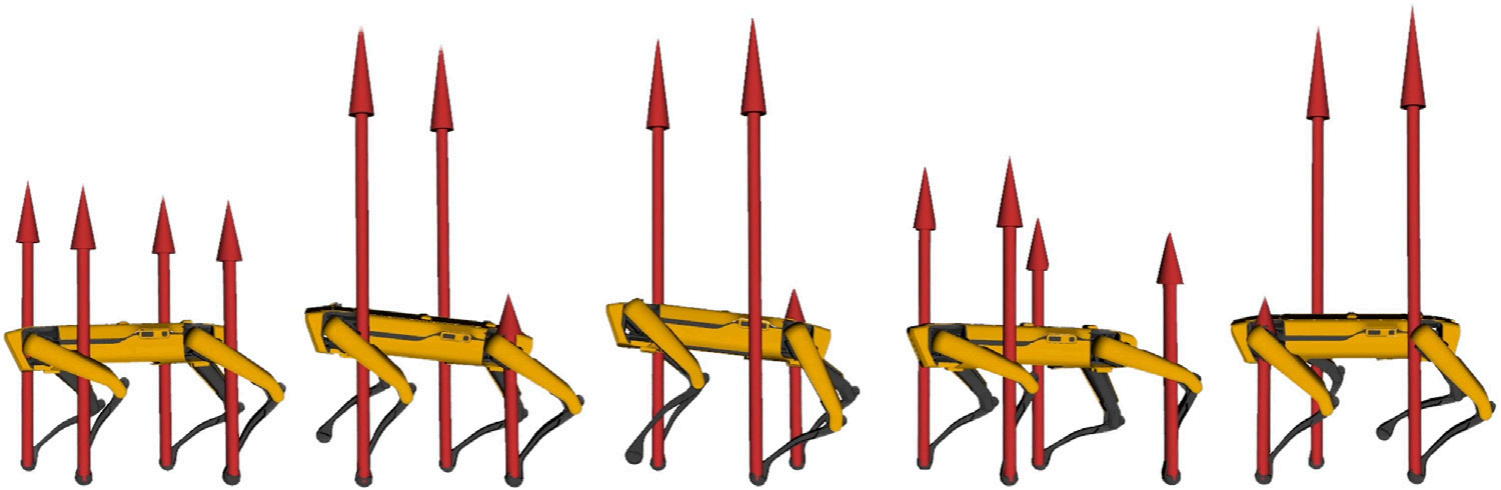
Frame sequence of Spot^®^ walking in a receding horizon fashion.

**FIGURE 11:**
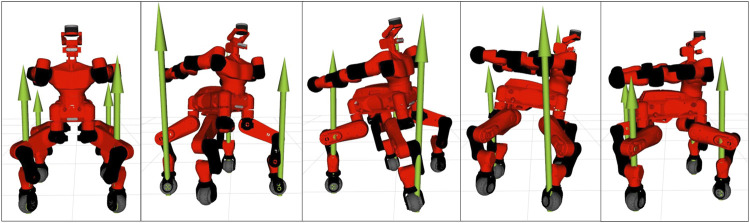
Frame sequence of the quadruped robot Centauro while performing a 90 deg in-place turn within two full gait cycles. The green arrows represent the contact forces at each foot.

**FIGURE 12:**
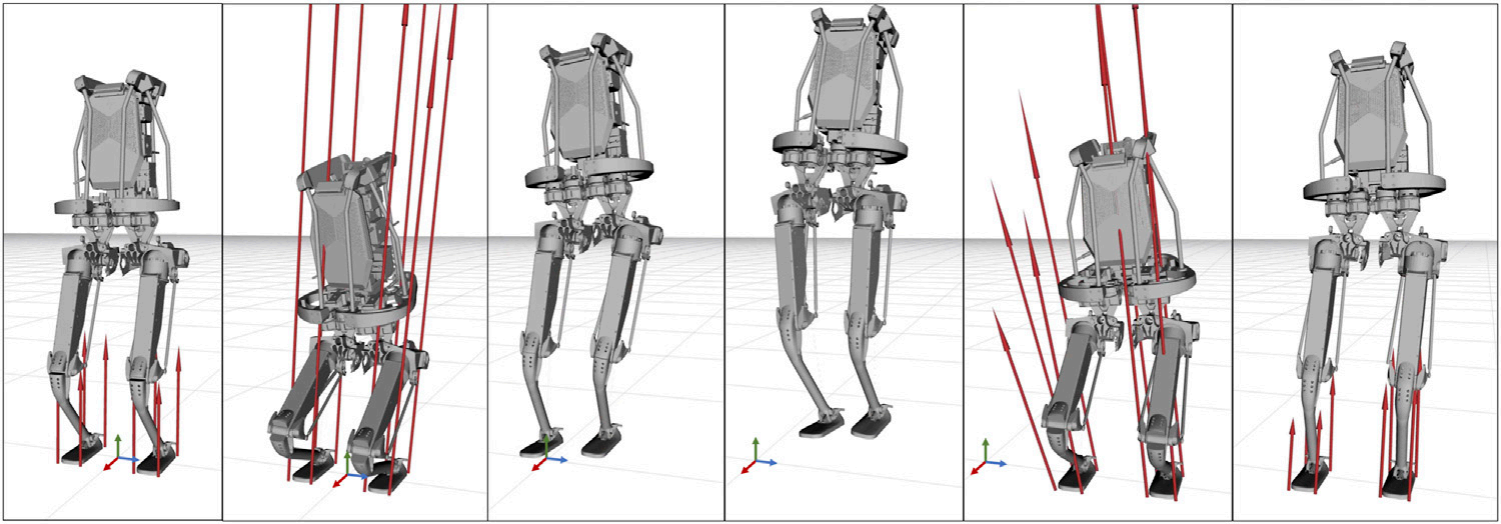
Frame sequence of the robot Kangaroo side-jumping. The red arrows represent the contact forces at each foot. Note that contact wrenches are parametrized in terms of four pure forces applied to the corners of each foot’s support area.

• Spot^®^ was used for a wide range of demonstrations, such as jumping, leaping (Figure 8, twisting (Figure 9) and raising on the hind legs. All these optimization problem follow the same structure: a contact schedule describes the manoeuvre in terms of contact phases, where either forces or contact velocities are constrained to zero. A final constraint fixes the last pose of the robot, similarly to the case study of Section 5. Horizon was also used for receding horizon applications, such as the generation of an endless walking motion (Figure 10). For this problem, besides the periodic contact schedule, a constant velocity reference is set as a cost function for the base link of the robot. Finally, a so-called *clearance constraint* imposes for each foot a fixed swing trajectory on the *z*-axis. The resulting problem was solved online with our ILQR solver.
• A crawling gait was successfully generated for the quadrupedal robot Centauro (Kashiri et al., 2019), both in simulation and on the real robot. Similarly to the previous applications, the optimal problem consists of a contact schedule and a final pose constraint. In particular, a forward and a turn-in-place crawl were generated via the ILQR solver, both exhibiting a comparatively large stride length as a consequence of the imposed gait pattern and final pose. A sequence of frames of the latter is shown in Figure 11.
• A set of jumping motions was generated for the robot Kangaroo from PAL Robotics^7^, as displayed in Figure 12. Furthermore, a side-to-side swing motion was obtained: by imposing a reference trajectory of the zero moment point (computed with the full robot dynamics), the biped shifts its weight from one foot to the other.
• The same swinging trajectory was obtained for the robot TALOS (Stasse et al., 2017), the full-size humanoid robot from PAL robotics.
• Dynamic trajectories were generated for a 2-DoF prototype robotic leg. Horizon proved to be useful for the initial design of a leg intended for agile motions. Given a set of tasks, the proposed framework was used to analyze the relevant parameters (such as maximum current, torque and angular velocity) in order to carry out the sizing of the motors.
• An industrial 7-DOF manipulator was used for a task-oriented optimization that minimizes the energy consumption while complying with the velocity limits that guarantee safety. Additionally, it includes in the optimization the position of the base link of the robot, demonstrating how Horizon can be successfully used for co-design applications and cell optimization, too.
• An optimal rappelling motion for a roped bipedal template was generated, mimicking the manoeuvres of aerial construction workers. In particular, the rope-assisted robot can extend/contract the rope and use the feet to jump from a vertical wall to which is connected trough the anchor-point of the rope. The rappelling motion consists in a controlled descent, obtained imposing a contact schedule and a final pose of the robot at the desired height after the vertical drop.

Clips for each application are gathered in the accompanying videos and at https://www.youtube.com/playlist?list=PL7c1ZKncPan72ef2Sof8Ky_TrlSK9qYYP. We finally invite the interested reader to further explore Horizon by visiting the repository at https://github.com/ADVRHumanoids/horizon.

## 6 Conclusion and Future Work

Trajectory optimization involving fast manoeuvres, physical interactions and geometric constraints using complex robot dynamics is a challenging field that has been tackled from many directions. Horizon aims at simplifying this process of generating and prototyping agile motions of robotic platforms without losing the performance that state-of-the-art algorithms can offer. To do so, it provides a flexible and intuitive pipeline that includes model description, NLP transcription and solvers. Its strength relies on the orchestration of advanced open-source tools, such as Pinocchio, CasADi, and ROS, to offer a reliable and fast computation to the user. To ensure compatibility with most applications, it supports standard description formats, and it is heavily customizable at almost all layers. Experimental results have demonstrated how, given a simple set of code instruction that translates into a NLP problem, complex motions (such as leaps, walking and rappelling) can be successfully generated by the optimization and manipulated for fast deployment. Horizon was tested on a vast selection of robots, spanning from simple template models such as the roped bipedal robot, or the cart-pole model, to full-dynamic models of quadrupedal robot of different complexity such as Spot^®^and Centauro, and humanoid robots such as TALOS and Kangaroo. Additionally, it was effectively used on prototype robotic legs and arms within co-design and cell optimization scenarios.

Nevertheless, Horizon currently lacks a well-developed control layer to close the loop on real hardware. Future work will extend the pipeline with a motion control layer that will open the path for MPC applications and robust robot deployment. Two other main research directions will be targeted: the first objective is providing the user with a set of tools for warm-starting the optimization problem, delving into the problem of motion memory as inspired by e.g. Mansard et al. (2018), where valuable initial guesses are stored in the form of a library of motions that can speed-up the optimization. The second line of research will explore path planning and environment interactions: adding a context for the robot to navigate, avoiding obstacles and breaking-establishing contacts with the surroundings towards some goal location. Furthermore, bigger variety of robots and skills will be tested to generate a database of off-the-shelf robot routines that can be integrated into desired applications. Finally, on the computational side, a range of enhancements is planned to offer a better support to code generation, as well as deployment to embedded devices.

## Figures and Tables

**FIGURE 13:**
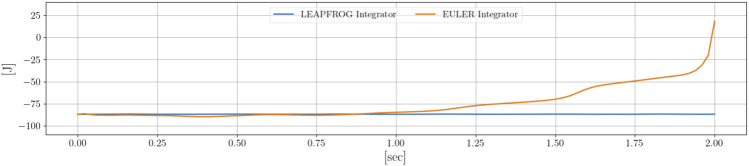
Comparison between the total energy of the system using the Leap-Frog and the Euler integrator. Using the Leap-Frog the energy is conserved, while the Euler method accumulates inaccuracies that incorrectly increase the energy of the system.

## Data Availability

The datasets presented in this study can be found in online repositories. The names of the repository/repositories and accession number(s) can be found below: https://github.com/ADVRHumanoids/horizon.

## Footnotes

2
www.mathworks.com.

3 Notice that if we consider the $\dot{\omega }$ expressed in the *local* frame of the floating base, Eq. 6 changes to $\dot{\rho }=\rho ◦\left[\frac{1}{2}\omega ,\;0\right]$ .

4 ILQR is also known as Differential Dynamic Programming (DDP), when second-order derivatives of the system dynamics are allowed.

5
http://wiki.ros.org/rviz.

6
http://wiki.ros.org/urdf.

7
https://pal-robotics.com/robots/kangaroo/.

## Appendix: LEAPFROG Integrator Application

Any ODE in *n* unknowns can be written in the general first-order form:

$$\dot{x}=f\left(x,u\right)$$
(34)

where **x** and **f** are *n*-component vectors. The so-called *Leap-Frog* is a second order accurate method which computes **x**_*k*+1_ as:

$$x_{k+1}=x_{k-1}+2\text{d}tf\left(x_{k},u_{k}\right)$$
(35)

Any *self-starting* scheme is required in order initialize the method by establishing the value of **x** at *k* = −1, e.g. by using Euler:

$$x_{-1}=x_{0}+\text{d}tf\left(x_{0},-u_{0}\right)$$
(36)

The Leap-Frog integrator is *time-reversible* and this property gives it some very important advantages as it guarantees conservation of energy and angular momentum for ODEs with periodic solutions Shampine (2009). Therefore, in situations where we are interested in long-term small changes in the properties of a nearly periodic orbit, and where even small systematic errors would mask the true solution, time-reversible integrators such as the Leap-Frog scheme are essential. As an example we consider a scenario where a simple template robot consisting in a box and two point-feet, is hanged by a rope and left free to swing from an initial angle. Assuming the rope mass to be negligible, we can approximate the period of the CoM oscillatory motion as:

$$T_{\text{CoM}}\approx 2\pi \sqrt{L/9.81}\approx 2.0\;s$$
(37)

where *L* ≈ 1.0 *m* is the distance of the CoM from the fixed point hanging the system. Thanks to the application of the Leap-Frog integrator, the total energy of the system remains practically unchanged during the whole control horizon. Using a different integrator than the Leap-Frog makes the problem diverge, as shown in Figure 13.
